# Identification of a vimentin-expressing α-cell phenotype in CF and normal pancreas

**DOI:** 10.1530/JOE-24-0190

**Published:** 2025-02-17

**Authors:** Nicole Kattner, Yan Hang, Nicole A J Krentz, Lydia A Russell, Matthew Palmer, Christine Flaxman, Nadine Plett, Rowan Coulthard, Yara Al-Selwi, Nicola Dyson, Minna Honkanen-Scott, Seung K Kim, Dina Tiniakos, Günter Klöppel, Sarah J Richardson, James A M Shaw

**Affiliations:** ^1^ Translational and Clinical Research Institute, Newcastle University, Newcastle upon Tyne, UK; ^2^ Department of Developmental Biology, Stanford University School of Medicine, Stanford, California, USA; ^3^ Stanford Diabetes Research Centre, Stanford University School of Medicine, Stanford, California, USA; ^4^ Faculty of Pharmaceutical Sciences, The University of British Columbia, Vancouver, British Columbia, Canada; ^5^ Islet Biology Group (IBEx), Exeter Centre of Excellence for Diabetes Research (EXCEED), University of Exeter College of Medicine and Health, Exeter, UK; ^6^ Department of Cellular Pathology, Royal Victoria Infirmary, Newcastle upon Tyne Hospitals NHS Foundation Trust, Newcastle upon Tyne, UK; ^7^ Department of Medicine (Endocrinology Division), Stanford University School of Medicine, Stanford, California, USA; ^8^ Department of Pediatrics (Endocrinology Division), Stanford University School of Medicine, Stanford, California, USA; ^9^ Department of Pathology, Aretaieion Hospital, Medical School, National and Kapodistrian University of Athens, Athens, Greece; ^10^ Institute of Pathology, Technical University of Munich, Munich, Germany; ^11^ Institute of Transplantation, Freeman Hospital, Newcastle upon Tyne Hospitals NHS Foundation Trust, Newcastle upon Tyne, UK

**Keywords:** diabetes, islet cells, histology, immunohistochemistry, inflammatory diseases

## Abstract

Endocrine dysfunction and diabetes can develop secondary to fibrotic diseases within the pancreas, including cystic fibrosis (CF). A phenotypic shift within epithelial cells has been recognised in association with pro-fibrotic signalling. We sought evidence of endocrine cell epithelial-to-mesenchymal transition in CF and non-CF pancreas. *Post-mortem* pancreatic sections from 24 people with CF and 10 organ donors without CF or diabetes were stained for insulin/glucagon/vimentin and Sirius red/fast green with collagen distribution assessed semi-quantitatively (CF) and quantitatively (non-CF). Analysis of existing single-cell RNA-sequencing datasets (three adult donors without diabetes and nine with chronic pancreatitis) for α-cell vimentin expression was performed. Cells co-expressing glucagon/vimentin were detected in a proportion (32(4,61)% (median (Q1,Q3))) of islets in all CF pancreata except donors dying perinatally. CF histopathology was characterised by peri-islet fibrosis, and 60(45,80)% of islets were surrounded by collagen strands. A positive correlation between islet fibrosis and vimentin-expressing α-cells was seen in non-CF donors <31 years (*r* = 0.972; *P* = 0.006). A possible association with donor age was seen in all donors (*r* = 0.343; *P* = 0.047). Single-cell RNA-sequencing analysis of isolated islets from non-diabetic donors and donors with chronic pancreatitis confirmed the presence of vimentin-positive and vimentin-negative α-cells. Differentiated α-cell function-associated gene expression was maintained. Differentially upregulated processes in co-expressing cells included pathways associated with extracellular matrix organisation, cell–cell adhesion, migratory capability and self-renewal. We have identified and characterised an intermediate epithelial/mesenchymal state in a sub-population of α-cells present throughout post-natal life, which may play a role in their response to extrinsic stressors, including fibrosis and ageing.

## Introduction

Cystic fibrosis (CF) is a hereditary disorder caused by mutations in the gene encoding the CF transmembrane conductance regulator (CFTR), impairing its function and leading to a build-up of viscous mucus in many organs, including the lungs, gut and pancreas (Shteinberg *et al.* 2021). In the pancreas, this is associated with progressive exocrine pathology, leading to pancreatic exocrine insufficiency (PEI) in 85% of people with CF (Löhr *et al.* 1989, Bogdani *et al.* 2017, Singh & Schwarzenberg 2017, Hart *et al.* 2018). CF also affects the endocrine pancreas with decreasing insulin secretion and abnormal α-cell function, eventually leading to the development of CF-related diabetes (CFRD) (Bridges *et al.* 2018, Nielsen *et al.* 2023). Diagnosis of diabetes not only adds to the treatment burden but also negatively impacts quality of life (Kwong *et al.* 2019). In addition, diabetes in CF is a risk factor for decreased pulmonary function, infections and poor nutritional status (Olesen *et al.* 2020).

Reduction in β-cell mass has been described in CFRD, but isolated islets from CF pancreata showed comparable stimulated insulin and glucagon secretion to control islets (Löhr *et al.* 1989, Bogdani *et al.* 2017, Cory *et al.* 2018, Hart *et al.* 2018). Increased relative α-cell mass has been reported in various studies (Löhr *et al.* 1989, Bogdani *et al.* 2017, Hart *et al.* 2018, Hull *et al.* 2018). Metabolic studies have consistently evidenced impaired β- and α-cell function in people with CF and PEI, compared to people with CF without PEI and people without CF (Moran *et al.* 1991, Sheikh *et al.* 2016, Nielsen *et al.* 2023). The underlying pathogenesis at a tissue level remains unknown (Norris *et al.* 2019, Umashankar *et al.* 2024). Several studies support low or absent expression of CFTR in human pancreatic endocrine cells, and given the relative sparing of overall islet mass even in most advanced disease, it is increasingly concluded that pancreatic exocrine pathology extrinsically drives endocrine dysfunction (Löhr *et al.* 1989, Couce *et al.* 1996, Bogdani *et al.* 2017, Cory *et al.* 2018, Hart *et al.* 2018, Di Fulvio *et al.* 2020, White *et al.* 2020).

Epithelial-to-mesenchymal transition (EMT) is a process common to pro-fibrotic pathology affecting a wide range of tissues (Deng *et al.* 2014, Liu *et al.* 2022), including CF lung disease (Quaresma *et al.* 2020, Mottais *et al.* 2023) and chronic pancreatitis (Deng *et al.* 2014).

We hypothesised that EMT may be evident in β- and α-cells in CF pancreata. To explore a true phenotypic shift at a structural protein level, we stained CF and non-CF pancreata with the classical mesenchymal marker vimentin, a type III intermediate filament core to the EMT process (Usman *et al.* 2021), to determine presence/absence in endocrine cells and explore potential spatial associations with pancreatic fibrosis. The expression of vimentin in pancreatic endocrine cells was further interrogated through single-cell RNA-sequencing (RNA-seq) analysis in donors without diabetes and with pancreatic fibrosis secondary to chronic pancreatitis. Vimentin was expressed in a proportion of α-cells within all post-natal pancreata studied with and without overt exocrine fibrosis (with the exception of a single donor with CFRD). Possible associations with age and fibrosis were identified, and positivity at a transcriptional level was associated with upregulation of extracellular organisation, cell migration and proliferation pathways.

## Methods

### Cohorts

Analysis included nine CF *post-mortem* pancreata (Klöppel cohort), ten CF *post-mortem* pancreata (Exeter Archival Diabetes Biobank (EADB)), five deceased donor pancreata with CFRD (Network of Pancreatic Organ Donors with Diabetes (nPOD)) and ten deceased donor pancreata without CF provided by the MRC Quality in Organ Donation (QUOD) Whole Pancreas Biobank (QUOD PANC). Non-CF donors had no previous diabetes diagnosis and no extensive pancreatic pathology, with tissue blocks obtained from the anterior body region (QUOD designation: P4A) (Kattner *et al.* 2021). Tissue blocks from CF pancreata were obtained *post-mortem* and biobanked by GK (Munich) and Dr Alan Foulis (Glasgow, EADB). Pancreatic tissue sections from five donors with confirmed CFRD were obtained from the nPOD biobank.

### Study approval

Work on this archival material is approved by the local ethics committee of the University Hospital ‘rechts der Isar’, Munich, Germany (document number: 281/19 s), and the West of Scotland Research Ethics Committee (ref: 20/WS/0074; IRAS project ID: 283620). Organs for the MRC QUOD Whole Pancreas Biobank were retrieved after written donor family consent in compliance with the UK Human Tissue Act of 2004, under specific ethical approvals by the UK Human Research Authority (05/MRE09/48 and 16NE0230).

### Histological staining and analysis

#### Chromogranin A / CD31 and Sirius red / fast green (SRFG) staining

Sectioning (4 μm thickness) followed by staining (CGA/CD31+SRFG) was performed by Novopath Laboratories at the Royal Victoria Infirmary, Newcastle upon Tyne Hospitals (NUTH). Double Chromogranin A (CGA) / CD31 immunostaining was performed using the Ventana Discovery Ultra TM (Roche Diagnostics, UK), following their optimised protocol. SRFG staining was performed, if possible, on sections serial to those used for CGA/CD31 staining, following their optimised protocol.

#### Evaluation of SRFG and immunohistochemical staining

SRFG and CGA/CD31 stained slides were scanned at 40× magnification at Newcastle Biobank (Leica Aperio AT2 (Leica Biosystems (UK) Ltd)), at Novopath Laboratories (Epredia P1000) or in Exeter (Akoya Biosciences PhenoImager Automated Quantitative Pathology Imaging System). SRFG-positive areas were assessed using DenseNet AI V2 and Area Quantification modules within the Indica Labs HALO Image Analysis Platform (version 3.2.1851.354). DenseNet AI V2 was used to classify islet and acinar areas, where islets were defined as groups of endocrine cells covering an area of ≥1,000 μm^2^. The area quantification module was used to define SRFG regions within the classified layers. SRFG area was calculated as a percentage of either the overall tissue section area or each of the tissue-classified areas. The peri-islet region was defined as an ∼33 μm halo around the islet, and the SRFG-positive area was calculated as a percentage of the total peri-islet area.

### Immunofluorescence staining and evaluation

Antigen retrieval was performed with sodium citrate buffer (pH 6) for 20 min at 100°C with cooling to 40°C. Tissue sections were incubated for 1 h with 20% fetal bovine serum (FBS) in phosphate-buffered saline (PBS) at room temperature, followed by incubation with primary antibodies overnight at 4°C. Primary antibodies were diluted in 0.05% FBS in PBS (Supplementary Table 1 (see section on Supplementary materials given at the end of the article)). ‘No primary antibody’ control sections diluted in 0.05% FBS in PBS were used for every donor in the GK / EADB cohorts and in a separate control section within each staining batch of nPOD donors. The following day, slides were washed three times in PBS for 5 min on a rotating platform, followed by incubation with secondary antibodies for 1 h at room temperature in darkness. Secondary antibodies were diluted in 0.05% FBS in PBS (Supplementary Table 1). Subsequently, slides were washed three times in PBS for 5 min on a rotating platform and incubated with 4′,6-diamidino-2-phenylindole (DAPI) solution (0.1 mg/mL in PBS) (Thermo Fisher, UK) for 20 min at room temperature in darkness. The slides were mounted with VECTASHIELD® Antifade Mounting Medium with DAPI (2BScientific, UK) and sealed with nail polish. All slides were stored at 4°C and protected from light before imaging on the Leica SP8 STED confocal microscope at the Bioimaging Unit at Newcastle University. Up to 50 islets per section were imaged and sectioned with a z-stack. Each image was assessed for insulin- and glucagon-positive cells and their potential co-expression of vimentin. If at least one α-cell expressed vimentin and glucagon, this islet was defined as positive for co-expression.

### RNA-seq analyses

For single-cell analyses of non-diabetic (ND) human islets, raw data were accessed from NCBI Gene Expression Omnibus, GSE196715 (Yoon *et al.* 2023). Data analysis was performed using the R package Seurat v.5.0.1 (Hao *et al.* 2023). Quality control steps included removing cells with more than 20% mitochondrial DNA and over 6,000 genes before SCT normalisation and integration. All cells were then clustered in UMAP space at a resolution of 0.6 with dimensions 1–40. Clusters were identified by marker gene expression, and endocrine cells (*INS*, *GCG* and *SST* transcript positive) were subsetted and re-clustered as above. A ridge plot was used to identify *VIM* high (>2.5 expression level) and *VIM* low (<0.5 expression level). Differential gene expression and gene ontology (GO) enrichment analyses were performed using *FindMarkers* and *DEenrichRplot*, respectively.

For the study of chronic pancreatitis islet cells, we reanalysed previously generated SmartSeq2-based single-cell RNA-seq results (Yan Hang JYL, Camunas-Soler J & Zhao W, unpublished observations). In brief, cells were obtained from nine subjects with chronic pancreatitis characterised by established exocrine fibrosis, including five without diabetes and four with pre-diabetes (HbA1c >39 mmol/mol (5.7%) and/or fasting glucose levels >5.6 mmol/L) (Supplementary Table 2). All analyses were performed using Seurat v3.1.1 (Stuart *et al.* 2019) following the developer’s manual (https://github.com/satijalab/seurat). Differential expression analysis of single-cell RNA-seq data was performed using the ‘model-based analysis of single-cell transcriptomics’ method as previously described (Finak *et al.* 2015). The *P* values were adjusted based on Bonferroni correction using all features remaining after QC filtering.

### Statistical analyses

Data are summarised as medians (Q1, Q3) or Pearson correlation. Pearson correlation and one-way ANOVA were performed with IBM SPSS Statistics, version 29.0.1.0 (171) (IBM, USA). Graphs were prepared with GraphPad Prism 9 (GraphPad Software, LLC, USA).

## Results

### Presence of vimentin-expressing α-cells in CF pancreata

To explore the potential of EMT in β- and α-cells in CF, triple immunofluorescence staining for insulin, glucagon and the mesenchymal marker vimentin was performed in 24 donor pancreata from three cohorts. These spanned an age range from perinatal death within the first week after birth to 33 years (Table 1).

**Table 1:** Summary of CF donor data.

Case ID	Sex	Age (years)	Percentage vimentin/glucagon co-expressing islets	Islets encircled by collagen (%)	CF status	Cohort
CF 1	Female	0	0	0.1	CF	GK
CF 2	Female	0	0	0.0	CF	GK
CF 3	Male	0.005479	0	44.9	CF	GK
CF 4	Male	0.333333	10	61.4	CF	GK
CF 5	NA	2	4	91.0	CF	EADB
CF 6	NA	3	4	86.3	CF	EADB
CF 7	NA	4	58	74.1	CF	EADB
CF 8	NA	4	30	56.7	CF	EADB
CF 9	Male	7	70	78.6	CF	GK
CF 10	NA	7	2	93.0	CF	EADB
CF 11	NA	7	90	86.5	CF	EADB
CF 12	NA	12	2	58.4	CFRD	EADB
CF 13	Female	13	74	55.0	CF	GK
CF 14	NA	14	34	84.5	CF	EADB
CF 15	NA	14	2	78.1	CFRD	EADB
CF 16	Male	19	52	74.1	CF	GK
CF 17	Male	19	48	44.1	CF	EADB
nPOD 6404	Female	21	68	91.3	CFRD	nPOD
CF 18	Male	27	16	14.8	CF	GK
CF 19	Male	27	38	48.5	CF	GK
nPOD 6136	Female	29	3	76.0	CFRD	nPOD
nPOD 6105	Female	31	0	43.1	CFRD	nPOD
nPOD 6202	Male	33	82	35.2	CFRD	nPOD
nPOD 6398	Male	33	16	53.3	CFRD	nPOD
**Median**		**13**	**16**	**60**		
**Q1, Q3**		**3.8, 22.6**	**2, 53.5**	**44.7, 80**		

Donor ID, sex, age, percentage of islets with vimentin/glucagon co-expression, percentage of islets encircled by collagen assessed by operator and origin of the sample are tabulated. Donors are arranged by increasing age at death. GK, Günter Klöppel cohort; EADB, Exeter Archival Diabetes Biobank; nPOD, Network for Pancreatic Organ Donors with Diabetes; NA, not available. Available RRID numbers: nPOD 6404 – SAMN15879457, nPOD 6136 – SAMN15879193, nPOD 6105 – SAMN15879162, nPOD 6202 – SAMN15879258 and nPOD – SAMN15879451.

**Figure 1:**
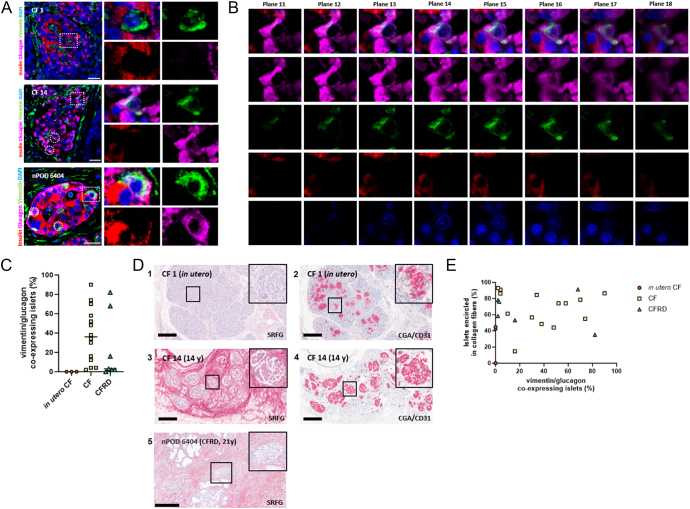
Vimentin expression within islets in human CF pancreata. (A) Immunofluorescence staining for insulin (red), glucagon (pink), vimentin (green) and DAPI (blue) in a donor who died at birth (CF 1, upper panels) with no evidence of vimentin expression in α- or β-cells, a donor dying at age 14 years (CF 14, middle panels) and a donor (aged 21 years) with confirmed CFRD (nPOD 6404, lower panel) with several vimentin and glucagon co-expressing cells. The squares indicate areas in magnified images. The circles indicate additional vimentin and glucagon co-expressing cells. Scale bar: 25 μm. (B) Confocal z-stack imaging to confirm co-localisation of vimentin and glucagon. The same frame of donor CF 14 in panel A showing planes 11–18. Merge (top row), glucagon (pink), vimentin (green), insulin (red) and DAPI (blue) are shown. (C) Percentage of pancreatic islets co-expressing vimentin and glucagon in donors with CF dying within the first week of life (*in utero* CF, *n* = 3); post-natal CF without known diabetes (CF, *n* = 14) and with confirmed CFRD (*n* = 7). (D) Exemplar images of SRFG staining (1) and CGA/CD31 staining (2) in an individual who died at birth (CF 1 (*in utero*)). Exemplar image of SRFG staining (3) and CGA/CD31 staining (4) in a CF donor (CF 14) aged 14 years (14 y). Exemplar image of SRFG staining (5) in a CFRD donor (nPOD 6404) aged 21 years (21 y). No CGA staining is available for this case. SRFG stains collagen red. Haematoxylin staining stains nuclei blue. CGA is in red; CD31 is in brown. The boxes indicate a magnified islet. Scale bar: 300 μm. (E) Vimentin and glucagon co-expressing phenotype versus peri-islet collagen. Percentage of vimentin/glucagon co-expressing islets plotted against percentage with uninterrupted peri-islet collagen assessed manually in SRFG histology images of CF donors (*in utero* CF (red circles), post-natal CF (yellow squares), confirmed CFRD (green triangles)). Pearson correlation coefficient for all CF donors: *r* = 0.227, *P* = 0.285; post-natal CF cases: *r* = −0.015, *P* = 0.959; and CFRD cases: *r* = −0.069, *P* = 0.882. A full-colour version of this figure is available at https://doi.org/10.1530/JOE-24-0190.

The presence of cells co-expressing glucagon and vimentin was demonstrated in CF pancreata by confocal microscopy (Fig. 1A), with expression of vimentin and glucagon within a single cell confirmed by z-stack imaging in all cases (Fig. 1B). With the exception of three individuals who died perinatally and one individual with CFRD aged 31 years, at least one co-expressing cell in at least 2% of islets examined was seen in all CF and CFRD donors (affecting median [Q1, Q3] 32 [4, 61]% islets) (Fig. 1C, Table 1). The proportion of islets containing α-cells with filamentous vimentin staining was highly variable in CF donors surviving beyond the first week of life in the presence (0–82% of islets) or absence (0–90% of islets) of known diabetes.

In contrast to this evidence supporting α-cell mesenchymal shift, no insulin and vimentin co-expressing cells could be identified in any islets of the CF donors.

We assessed collagen distribution in all CF pancreata following SRFG staining. Inter- and intra-lobular stroma, including collagen strands both within and around islets, were observed in pancreata from donors dying perinatally (Fig. 1D1 and 2). Older donors were characterised by progressive fibrosis associated with increasing acinar atrophy and ultimately adipocyte replacement (Fig. 1D3, 4 and 5). Cases with known CFRD exhibited both fibrotic (example image in Fig. 1D5) and adipocyte replacement phenotypes. Islets identified by CGA staining in serial sections remained present, clustered together and surrounded by residual fibrotic bands even after diabetes diagnosis (Fig. 1D5) and after replacement of virtually all of the parenchyma with fat (Löhr *et al.* 1989). Identification of endothelial cells by CD31 staining in these *post-mortem* donors with CF was more challenging. Maintained peri- and intra-islet vasculature in younger individuals was confirmed (Fig. 1D2), with reduced vascularity in older CF donors as previously reported (Castillo *et al.* 2022, Malik *et al.* 2023).

The heterogeneous patterns of collagen distribution in combination with severe overall tissue pathological disorganisation made quantitative analysis of tissue morphology challenging. Semi-quantitative assessment of the proportion of islets surrounded by an uninterrupted matrix of collagen fibres using the SRFG slides was performed. This revealed an extensive range in the proportion of islets encircled by collagen strands across donors (0–93%), with median 60 (5, 80)% affected (Table 1, Fig. 1E).

**Figure 2:**
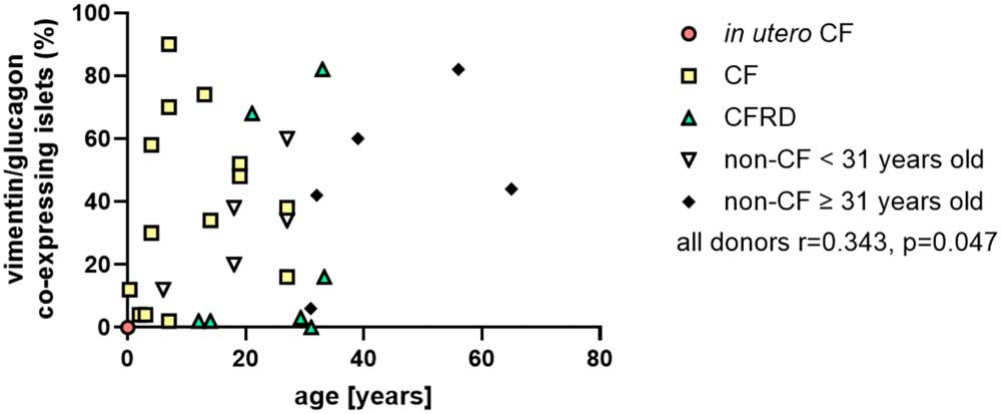
Vimentin and glucagon co-expressing phenotype versus age. Percentage of vimentin and glucagon co-expressing islets plotted against age in CF and non-CF donors (*in utero* CF (red circles), post-natal CF without diabetes (yellow squares), confirmed CFRD (green triangles), control donors aged <31 years (white triangles) and control donors aged 31 years or older (black diamonds)). Pearson correlation coefficient for all donors: *r* = 0.343, *P* = 0.047; all CF cases: *r* = 0.187, *P* = 0.380; CF cases without diabetes: *r* = 0.149, *P* = 0.611; CFRD cases: *r* = 0.272, *P* = 0.555; and non-CF cases without diabetes: *r* = 0.608, *P* = 0.062. A full-colour version of this figure is available at https://doi.org/10.1530/JOE-24-0190.

In this CF cohort with or without diabetes, there was no significant association between the percentage of islets containing vimentin/glucagon-co-expressing cells and the proportion surrounded by uninterrupted collagen (Fig. 1E) or donor age (Fig. 2).

### Presence of vimentin-expressing α-cells and islet fibrosis in non-CF pancreata

**Figure 3:**
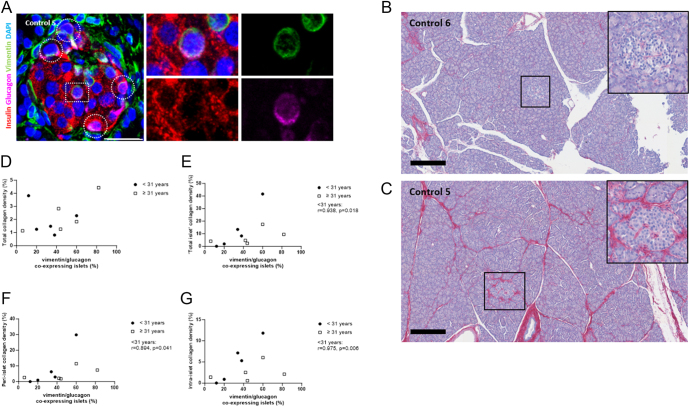
Vimentin expression in non-CF human islets. (A) Immunofluorescence staining of one example islet of control 6 showing insulin (red), glucagon (pink), vimentin (green) and DAPI (blue). The circles indicate cells with glucagon and vimentin co-localisation. The square denotes a magnified area. Scale bar: 25 μm. (B + C) SRFG staining of non-CF donor 6 (B) and non-CF donor 5 (C) with low (non-CF 6) and higher (non-CF 5) collagen density in the whole section, in addition to the peri-islet and intra-islet area shown at higher magnification in the boxes. All collagen is stained in red, with haematoxylin used as a counterstain. Scale bar: 300 μm. (D, E, F and G) Plots of the percentage of islets with altered α-cell phenotype versus percentage collagen density. Overall collagen percentage within section (D), total islet collagen (peri-islet + intra-islet) (E), peri-islet (F) and intra-islet (G) collagen density compared with the percentage of islets with vimentin/glucagon co-expressing α-cells in control donors <31 years old (*n* = 5; black circles) and ≥31 years old (*n* = 5; white squares). Pearson correlation coefficients: (D) all: *r* = 0.397, *P* = 0.256; <31 years: *r* = −0.31, *P* = 0.612; ≥31 years: *r* = 0.766, *P* = 0.131. (E) All: *r* = 0.519, *P* = 0.125; <31 years: *r* = 0.938, *P* = 0.018; ≥31 years: *r* = 0.543, *P* = 0.344. (F) All: *r* = 0.536, *P* = 0.111; <31 years: *r* = 0.894, *P* = 0.041; ≥31 years: *r* = 0.611, *P* = 0.273. (G) All: *r* = 0.432, *P* = 0.213; <31 years: *r* = 0.975, *P* = 0.006; ≥31 years: *r* = 0.356, *P* = 0.557. A full-colour version of this figure is available at https://doi.org/10.1530/JOE-24-0190.

We performed insulin/glucagon/vimentin and SRFG staining in sections from ten organ donors without CF, diabetes or established chronic pancreatitis. There was evidence of glucagon-positive cells co-expressing vimentin (Fig. 3A) in at least 6% of islets in all donors studied, with median 40 [23.5, 56]% affected (Table 2).

**Table 2:** Summary of non-CF donor data.

Donor ID	Sex	Age (years)	Percentage vimentin/glucagon co-expressing islets	Percentage whole section collagen	Percentage ‘total islet’ collagen	Percentage peri-islet collagen	Percentage intra-islet collagen
Non-CF 1	Female	6	12	3.82	0.04	0.03	0.02
Non-CF 2	Female	18	20	1.25	1.88	0.99	0.89
Non-CF 3	Female	18	38	0.81	8.27	2.96	5.31
Non-CF 4	Male	27	34	1.48	13.42	6.28	7.14
Non-CF 5	Female	27	60	2.29	41.64	29.81	11.83
Non-CF 6	Male	31	6	1.13	4.06	2.65	1.41
Non-CF 7	Male	32	42	2.83	4.63	2.11	2.52
Non-CF 8	Female	39	60	1.83	17.47	11.43	6.04
Non-CF 9	Female	56	82	4.43	9.49	7.37	2.12
Non-CF 10	Female	65	44	1.26	2.41	1.83	0.58
**Median**		**29**	**40**	**1.7**	**6.5**	**2.8**	**2.3**
**Q1, Q3**		**20, 37**	**23.5, 56**	**1.3, 2.7**	**2.8,12.4**	**1.9, 7.1**	**1.0, 5.9**

Donor ID, sex, age, percentage of islets with vimentin-positive α-cells, percentage of collagen in the whole section, percentage of collagen in the total islet area consisting of peri- and intra-islet area, percentage of collagen in the peri-islet area and percentage of collagen in the intra-islet area. Collagen density was assessed by AI. Donors are arranged by increasing age. All donors originate from the QUOD Whole Pancreas tissue bank at Newcastle University.

SRFG staining revealed a wide range of patterns and densities of collagen distribution, from non-pathological structural collagen (Fig. 3B) to abnormal inter- and intra-lobular collagen deposition, including peri-islet and intra-islet fibrosis (Fig. 3C). The less disrupted tissue composition in these donors enabled the use of a deep learning classification tool (Indica HALO AI) to quantify collagen density in whole tissue sections, where ‘total islet’ area (peri- plus intra-islet region), peri-islet area (the extra-islet region extending 33 μm outside of the islet border) and intra-islet (within the islet border) area were assessed (Table 2, Fig. 3D, E, F and G). In this non-CF cohort, no association was seen between collagen density within the overall section and the proportion of vimentin- and glucagon-co-expressing cells (Fig. 3D). Significant correlations were, however, seen between intra-islet collagen density and the putative α-cell EMT phenotype in the age-matched donors to the CF cohort (donor age <31 years: intra-islet collagen vs vimentin-positive α-cells; *r* = 0.972; *P* = 0.006; Fig. 3E, F and G).

A trend towards a correlation between donor age and the proportion of islets containing vimentin-positive α-cells was seen in donors without CF or diabetes (*r* = 0.608; *P* = 0.062; Fig. 2) and in all donors studied with and without CF (*r* = 0.343; *P* = 0.047).

### Single-cell RNA-seq analysis of donors without diabetes or CF

**Figure 4:**
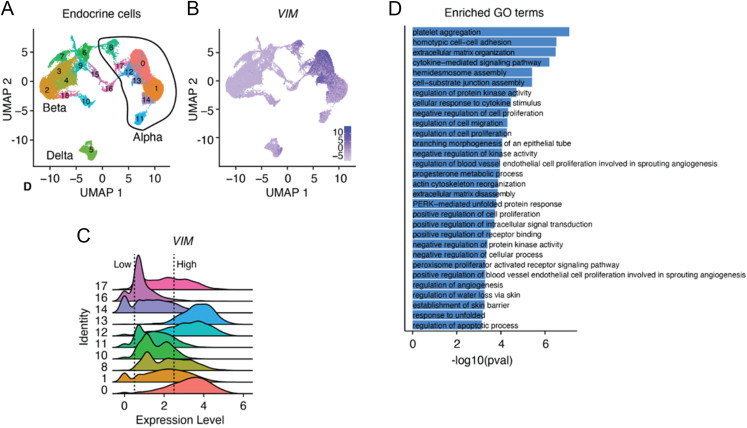
VIM+ α-cells in human donors with diabetes express EMT-associated genes. (A) UMAP of islet endocrine cells from three human donors. (B) UMAP of VIM transcript expression. (C) Ridge plot of VIM gene expression in α-cell clusters. (D) Enriched GO terms in VIM-high (>2.5 expression level) compared to VIM-low (<0.5 expression level) α-cells. A full-colour version of this figure is available at https://doi.org/10.1530/JOE-24-0190.

We analysed a publicly available single-cell transcriptomics dataset of pancreatic islet endocrine cells isolated from three deceased donors (male, 54–57 years old) without diabetes or CF (Fig. 4A) (Yoon *et al.* 2023). *Vimentin* (*VIM*) transcript was detected in several of the α-cell clusters but was rarely detected in β- and δ-cells (Fig. 4B). Within α-cells, *VIM* transcript expression was variable, including clusters with bimodal expression (Fig. 4C). To understand the biological processes that are enriched in vimentin-positive α-cells, we performed differential gene expression analysis on α-cells with high (>2.5 expression level) versus low (<0.5 expression level) *VIM* gene expression. This revealed 185 upregulated and 122 downregulated genes in vimentin-expressing α-cells (Supplementary Table 3). Enriched GO terms for the high vimentin-positive α-cells included ‘cell–cell adhesion’, ‘extracellular matrix organization’, ‘cellular response to cytokine stimulus’, ‘regulation of cell proliferation’ and ‘regulation of cell migration’ (Fig. 4D). Expression of genes associated with normal α-cell function was maintained without significant loss of E-cadherin gene expression or any difference in expression of classical EMT-associated genes, including *CDH2* (N-cadherin), *FN1* (fibronectin), *ACTA2* (alpha smooth muscle actin), *TWIST1*, *SNAI1* (snail), *SNAI2* (slug), *ZEB1/2* and *FOXC2*.

### Single-cell RNA-seq analysis of chronic pancreatitis

**Figure 5:**
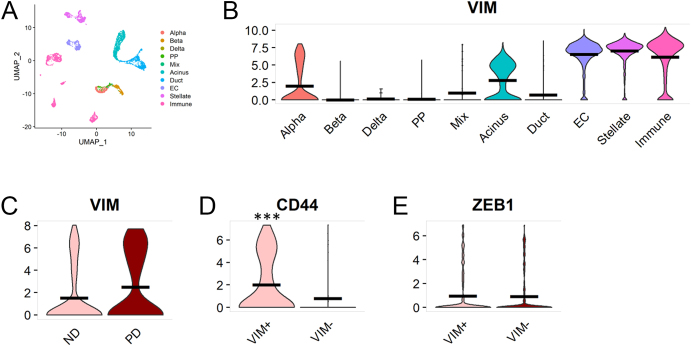
Vimentin (VIM) gene expression in endocrine cells isolated from nine human donors with chronic pancreatitis. (A) UMAP of all cell types from nine human donors with chronic pancreatitis. (B) Expression of vimentin in all endocrine and exocrine cell types. (C) Expression of vimentin in α-cells of ND (*n* = 5) and PD (*n* = 4) donors with chronic pancreatitis. PD was defined as HbA1c >39 mmol/mol (5.7%) and/or fasting blood glucose >5.6 mmol/L. (D) Expression of CD44 in VIM-positive vs VIM-negative α-cells. (E) Expression of ZEB1 in VIM-positive vs VIM-negative α-cells. PP: pancreatic polypeptide; EC: endothelial cells. ***P <0.01 vs VIM-negative α-cells. A full-colour version of this figure is available at https://doi.org/10.1530/JOE-24-0190.

Single-cell analysis of dissociated pancreatic cells from nine living donors with chronic pancreatitis confirmed vimentin gene expression in pancreatic stellate, immune, endothelial and acinar cells (Fig. 5A and B). In the endocrine compartment, vimentin transcript was detected in a distinct sub-population (39%) of α-cells, although no significant expression in β-, δ- or pancreatic polypeptide cells was observed (Fig. 5B). Dividing donors into those without diabetes and those with pre-diabetes (HbA1c >39 mmol/mol (5.7%) and/or fasting blood glucose >5.6 mmol/L)) revealed a higher proportion of vimentin-positive α-cells in pre-diabetic (PD) (46%) than in non-diabetic (ND) (32%) donors (Fig. 5C).

Expression of core phenotypic markers, including glucagon, *ARX*, *MAFB* and chromogranin A, was comparable in vimentin-positive and vimentin-negative α-cells (Table 3). Twenty-two genes were significantly enriched in vimentin-positive compared with vimentin-negative α-cells (false discovery rate (FDR) <0.05, Table 3, Supplementary Table 4). These included genes involved in cancer progression (*PDK4*, *GPX3*, *LMAN1*, *RP11-124N14.3* and *PAK6*), factors implicated in cytokine response (*TNFRSF12A*, *UBE2J1 and MAP1B*), as well as those important for cytoskeleton remodelling, decreased intercellular adhesion and cancer stem cell transformation (*EZR* and *CD44*). An example violin plot for CD44 is shown in Fig. 5D. Other significantly upregulated genes were largely driven by a single case. *ZEB1* (Fig. 5E) and other classical EMT-associated markers (including *ZEB2*, *TWIST1*/*2*, *SNAI1*, *SNAI2*, *SLUG*, *FN1*, *ACTA2*, *CDH2* and *FOXC2*) were not significantly upregulated. Four genes (*TNFRSF12A*, *MAP1B*, *EZR* and *YWHAZ*) upregulated in donors with chronic pancreatitis were also upregulated in vimentin-positive α-cells in donors without diabetes or CF (Supplementary Table 3). No genes were significantly downregulated in this analysis, in keeping with maintenance of epithelial cell phenotype and α-cell function-associated genes, in parallel with upregulation of the above genes potentially associated with motility, self-renewal and resilience to cytokine-mediated stress. GO analysis did not yield meaningful data in view of the small number of differentially expressed genes.

**Table 3:** List of differentially expressed genes in vimentin-positive α-cells compared to vimentin-negative α-cells.

Alpha-cell					Cancer-associated					Cytokine response				
Gene	Average logFG	Pct.1	Pct.2	Adjusted *P*-value	Gene	Average logFG	Pct.1	Pct.2	Adjusted *P*-value	Gene	Average logFG	Pct.1	Pct.2	Adjusted *P*-value
*GCG*	0.4637	1.000	0.991	1	*PDK4*	1.4234	0.763	0.414	1.77E-09	*TNFRSF12A*	1.2005	0.674	0.391	0.0005
*ARX*	−0.4993	0.459	0.409	1	*GPX3*	0.9084	0.867	0.740	0.0007	*UBE2J1*	0.3772	0.756	0.456	0.0065
*MAFB*	−0.4953	0.607	0.647	1	*LMAN1*	0.4880	0.785	0.474	0.0013	*MAP1B*	0.6232	0.933	0.842	0.0255
*CHGA*	−0.1838	1.000	0.995	1	*RP11-124N14.3*	0.1096	0.119	0.000	0.0018					
					*PAK6*	0.9423	0.556	0.260	0.0061					
					*HBB*	0.2976	0.289	0.074	0.0313					

Metabolism					Cytoskeleton/stemness					Others				
Gene	Average logFG	Pct.1	Pct.2	Adjusted *P*-value	Gene	Average logFG	Pct.1	Pct.2	Adjusted *P*-value	Gene	Average logFG	Pct.1	Pct.2	Adjusted *P*-value
*SLC2A13*	0.6135	0.600	0.293	0.0031	*EZR*	0.6808	0.948	0.814	0.0045	*TRBV13*	0.6282	0.326	0.088	0.0003
*YWHAZ*	0.5258	0.978	0.902	0.0245	*CD44*	0.9954	0.511	0.219	0.0056	*TRBV11-3*	0.8068	0.326	0.088	0.0006
*PDZD8*	0.5475	0.807	0.553	0.0357						*TRBV18*	0.8748	0.326	0.088	0.0013
										*TRBV16*	0.7611	0.326	0.088	0.0019
										*TRBV12-5*	0.9439	0.341	0.093	0.0024
										*TRBV17*	0.9335	0.326	0.093	0.0157
										*TRBV7-9*	0.4960	0.304	0.088	0.0319
										*IGLV3-27*	0.2120	0.274	0.070	0.0344

Pct.1, fraction of vimentin-positive α-cells expressing the specific genes. Pct.2, fraction of vimentin-negative α-cells expressing the specific genes.

## Discussion

Using immunofluorescence co-staining and single-cell RNA-seq analysis, we have demonstrated co-expression of the classical mesenchymal marker vimentin in a proportion of glucagon-positive pancreatic α-cells in *post-mortem* donors with CF. This phenotype was also present in a comparator deceased donor cohort without clinically evident pancreatic endocrine or exocrine pathology. In this cohort, the proportion of islets, including glucagon- and vimentin-co-positive cells, correlated with the extent of islet fibrosis in donors under 31 years of age and a trend towards correlation with donor age was seen. Genes expressed more abundantly in these α-cells are associated with response to cytokine-induced stress, proliferation and migration.

Alpha-cells staining positive for the filamentous protein vimentin were initially identified in triple immunofluorescence co-staining studies in a *post-mortem* cohort with CF. This phenotype was present in relatively few cells per affected islet but in up to 90% of islets. Interestingly, only three individuals dying within 2 days of birth and a single older individual with confirmed CFRD had no evidence of vimentin-positive α-cells. In individuals with CF living beyond the first week of life, there was a wide variability in the percentage of islets, including vimentin protein-expressing cells in those with and without known diabetes. SRFG staining confirmed the presence of peri-islet fibrosis throughout the course of post-natal CF disease progression. The severity of tissue disorganisation precluded accurate quantification of fibrosis using AI-based tissue classification, and semi-quantitative assessment of islets surrounded by fibrosis in CF by visual assessment was not significantly associated with the presence of vimentin and glucagon co-positivity.

A reduction in acinar mass development *in utero* reflected by an increased ratio of connective tissue to acinar tissue area has been reported in a previous quantitative pathological comparison of early post-natal CF versus non-CF (Imrie *et al.* 1979). In that study, it was concluded that this reflected a lack of normal exocrine pancreas maturation or persistence of an earlier fetal-stage phenotype, but that degenerative changes did not ensue until after birth (Imrie *et al.* 1979). Similarly, in a porcine CF model, despite fetal pancreatic abnormalities, overt fibrosis associated with increased expression of the pivotal EMT signalling molecule TGF-β1 did not occur until post-natal life (Abu-El-Haija *et al.* 2012). This provides a potential rationale for the absence of evidence for vimentin-positive α-cells at birth in the current study and supports the hypothesis that this phenotypic shift may be induced by stresses associated with exocrine pancreatic fibrosis. The number of *in utero* CF cases studied was small, however, and may not be truly representative of this population.

Although no significant associations between α-cell vimentin expression and the extent of peri-islet fibrosis or donor age were demonstrated in CF in the current study, extensive fibrotic changes were seen in all donors surviving beyond birth.

Vimentin-expressing α-cells were also present in a proportion of islets in all cases within a comparator cohort of deceased organ donors without diabetes or clinically evident pancreatic exocrine disease. The absence of advanced pathology within this cohort enabled quantitative assessment of fibrosis by AI-based tissue classification. A possible association of altered α-cell phenotype with donor age was seen. Vimentin expression in α-cells was not associated with overall pancreatic fibrosis, but there was a significant correlation with the extent of intra-islet fibrosis in younger donors.

EMT evidenced by vimentin expression potentially associated with TGF-β signalling is believed to play a role during endocrine cell development *in utero*, enabling delamination from the trunk epithelium and subsequent migration to form proto-islets (Bastidas-Ponce *et al.* 2017). In early rat and mouse pancreas development, glucagon and vimentin co-staining has been reported in almost all glucagon-positive cells but no β-cells or other endocrine precursor cells. The proportion of co-expressing cells decreased over time, with few present at the end of normal gestation (Bella *et al.* 2009).

Positive co-staining with glucagon and vimentin has been reported in isolated case reports in biopsies from patients with chronic pancreatitis (Beamish *et al.* 2019) and type 2 diabetes (White *et al.* 2013). In a single previously published case series (Roefs *et al.* 2017), vimentin/glucagon and vimentin/insulin co-staining were seen in deceased organ donors with and without type 2 diabetes, with higher numbers of co-expressing α-cells than β-cells and higher numbers of both altered phenotypes in diabetes. Electron microscopy with immunogold staining demonstrated the presence of vimentin in intermediate filament bundles within the cytoplasm of α-cells in type 2 diabetes (Roefs *et al.* 2017). In β-cells, however, vimentin immunostaining was primarily in lysosomes (Roefs *et al.* 2017). In the current study, co-expression with vimentin could not be identified in any insulin-positive cells in donors with or without CF.

In the Roefs *et al.* study, EMT associated with type 2 diabetes was postulated as the pathological mechanism inducing expression of the mesenchymal marker vimentin in pancreatic endocrine cells (Roefs *et al.* 2017). The youngest donor without diabetes in that series was 27 years' old, and the youngest donor with diabetes was 34 years' old. The current study focused on younger donors, providing convincing evidence of the expression of vimentin in a small proportion of islet α-cells throughout post-natal life but no evidence of vimentin-expressing β-cells. We have previously proposed that vimentin expression in β-cells in type 2 diabetes represents loss of end-differentiation associated with loss of function (White *et al.* 2013, White *et al.* 2016). The current unexpected finding of vimentin-expressing α-cells in pancreata of healthy young individuals suggested to us that this may constitute a more transient, reversible phenotypic state as opposed to the loss of cellular identity suggested in previous publications (Roefs *et al.* 2017).

Recent consensus guidelines have recommended the term ‘epithelial-mesenchymal-plasticity’ (EMP) to describe a cell type’s ability to transition along an epithelial-to-mesenchymal spectrum, adopting ‘hybrid epithelial/mesenchymal’ states expressing epithelial features and mesenchymal characteristics, including migratory capability (Yang *et al.* 2020). In the current study, vimentin staining was in a classical filamentous pattern associated with some loss of classical endocrine cell circularity. This has been described in carcinogenesis as ‘early hybrid EMP’, potentially enabling tissue remodelling and self-renewal, with cancer cells becoming more elongated in the ‘late hybrid’ stage with suppression of epithelial phenotype and complete loss of cell adhesion (Usman *et al.* 2021). We further validated our finding of a sub-population of vimentin-expressing α-cells in donors with and without overt fibrotic exocrine pathology by interrogating single-cell RNA-seq databases derived from dissociated islets from normal donors and donors with chronic pancreatitis. Vimentin-expressing α-cells were confirmed in both datasets. The presence of core phenotype-defining genes, including *ARX* and *MAFA*, in addition to glucagon, was confirmed in vimentin-positive cells, with comparable expression levels to vimentin-negative cells. Alpha-cell EMT in post-natal life has recently been proposed through single-cell RNA-seq in donors encompassing a wide age range (18 days to 65 years old) (Avrahami *et al.* 2020). It was proposed that this was associated with relative immaturity in α-cells versus β-cells, with expression of mesenchymal markers but maintained expression of core epithelial markers, including E-cadherin. Adult α-cells are known to have a significantly higher proliferation rate than β-cells, and the authors concluded that this may be facilitated by EMT enhancing response to mitogenic signals and migratory capacity. Relative upregulation of genes associated with EMT was reported in α-cells isolated and sequenced from donors with type 2 diabetes, in addition to TGF-β signalling pathway genes, including TGF-β receptor 1. TGF-β signalling is a central driver of EMT in fibrotic disease, and we have shown increased TGF-β1 expression in pancreata within the current CF cohort in comparison with non-CF control donors (unpublished data).

In the current analysis, vimentin-positive α-cells in ND control islets without evident pancreatic pathology show enriched GO terms for cell–cell adhesion, ECM organisation, response to cytokine stimuli, migration and proliferation. Transcription factors classically associated with definitive EMT, such as *TWIST*, *ZEB* and *SNAI1*, (Ichikawa *et al.* 2022) were not upregulated. Epithelial phenotype gene expression, including E-cadherin, was maintained.

In the absence of available single-cell RNA-seq data from donors with CF, we further confirmed the presence of both vimentin-positive and vimentin-negative α-cell sub-populations in donors with chronic pancreatitis characterised by established fibrosis. Chronic pancreatitis shares common pathological features with CF, and similar mechanisms through which exocrine disease may affect endocrine cell phenotype and function have been proposed (Klöppel *et al.* 2004, Rickels *et al.* 2020). In cells from donors with chronic pancreatitis, there was a limited set of 22 differentially expressed genes in vimentin-positive α-cells, likely due to the limited scale of the dataset. The differentially expressed genes supported the hypothesis of a mesenchymal shift evidenced by expression of the mesenchymal cytoskeletal protein vimentin in response to extracellular environment signals, including potentially peri-islet fibrosis. As in the ND donors, this shift was not associated with upregulation of the genes classically associated with ‘binary’ EMT in scarring fibrosis or tumorigenesis. Many of the upregulated genes in vimentin-expressing α-cells in chronic pancreatitis are associated with motility and proliferation in epithelial cancer cells expressing mesenchymal markers (Anwar *et al.* 2021). For example, PAK6 promotes cell–cell disadhesion and PDK4 is associated with cell detachment from extracellular matrix (Gong *et al.* 2020). CD44 is a transmembrane glycoprotein providing a connection between the cytoskeleton and the extracellular matrix, playing a key role in cellular interactions with their niche. It is a canonical marker of cancer stem cells upregulated during a mesenchymal shift, conferring proliferative capability, survival and resistance to apoptosis (Xu *et al.* 2015). EZR is also a cancer stem cell marker associated with decreased intercellular adhesion and increased motility (Ahmed *et al.* 2022). In keeping with previous reports of increased α-cell vimentin gene and protein expression in type 2 diabetes (Roefs *et al.* 2017, Avrahami *et al.* 2020), a greater proportion of α-cells were vimentin-expressing in the chronic pancreatitis donors with pre-diabetes.

This study has several limitations. Our analysis was dependent on archival collections of relatively young *post-mortem* donors with CF. While autolysis between death and tissue fixation may have impacted tissue morphology and antigen preservation, high-quality standard and immunofluorescence staining was achieved. Highly disrupted tissue organisation due to severe pancreatic exocrine pathology precluded truly quantitative AI-augmented analysis of islet fibrosis in CF. Semi-quantitative assessment of peri-islet fibrosis was further complicated by increased collagen-rich pancreatic stroma in younger donors with a relatively low proportion of vimentin-expressing α-cells – potentially explained by the paucity of pathological ‘scarring’ fibrosis at this stage of disease pathogenesis. AI-augmented quantification of fibrosis in the donors without CF enabled clearer identification of associations within the novel phenotype. Identification of vimentin-expressing α-cells was dependent on microscopic confirmation of the presence of both immunostains within single cells. Confidence was increased through confocal z-stack analysis. In both CF and non-CF cohorts, a comparison of the percentage of islets containing vimentin-positive α-cells and the percentage with peri-islet fibrosis was made by immunofluorescence co-staining and SRFG collagen staining on separate sections from the same tissue block. This enabled donor ‘block-level’ associations to be determined but not confirmation of whether or not individual islets containing vimentin-positive α-cells were more likely to be surrounded by fibrosis.

Reliance on historical samples precluded parallel single-cell RNA analysis in CF donors. Our findings were, however, supported by analysis of a published dataset and sequencing of dissociated islet cells from live donors with pancreatic exocrine fibrosis secondary to chronic pancreatitis. Due to the intrinsic differences between the deceased donor and live resection chronic pancreatitis cohorts, including cold ischaemic times and differences between cell isolation/dissociation protocols and the use of different single-cell approaches (10× genomics and SmartSeq-2) to generate the datasets, we were not able to directly compare gene expression levels between normal donors and those with chronic pancreatitis (Gloyn *et al.* 2022). We were, nonetheless, able to confirm highly comparable upregulated pathways, further supporting the presence of this hybrid epithelial/mesenchymal state in health and disease. In addition, we identified a more clearly separate vimentin-positive population in chronic pancreatitis, with a higher proportion of these cells in those with pre-diabetes.

Our data regarding potential associations of vimentin expression in pancreatic α-cells with age and fibrosis, together with the signalling pathway drivers/functional impact of this phenotype, remain only hypothesis-generating. Nevertheless, confirmation (by immunofluorescence co-staining and scRNA-seq) of its presence in a proportion of α-cells throughout post-natal life, in contrast to β-cells, underlines the need for further studies designed to elucidate underlying mechanisms.

In conclusion, we have identified an α-cell type expressing the mesenchymal marker vimentin in CF pancreas together with an association with the extent of islet fibrosis in non-CF donors. Vimentin expression was not seen in β-cells in these cohorts but was identified in a population of α-cells in the majority of donors with and without CF beyond the first week of post-natal life. The drivers of this phenotypic heterogeneity within α-cells, together with the roles and function of vimentin-positive cells, remain unclear, although upregulated genes in these cells suggest the potential that this may be associated with the apparent greater ‘resilience’ in the face of stress and ageing in human α-cells through an enhanced ability to survive, migrate and self-renew in comparison to β-cells.

## Supplementary materials



## Declaration of interest

The authors declare that there is no conflict of interest that could be perceived as prejudicing the impartiality of the work.

## Funding

This project was supported by the Cystic Fibrosis Trust SRC 019, the MRC (Quality and Safety in Organ Donation Tissue Bank – Expansion to include Pancreas/Islets, Heart and Lungs) (MR/R014132/1) and the Ray Wilson Memorial Fund (Newcastle University). We acknowledge the Diabetes Genomics and Analysis Core and the Stanford Islet Research Core of the Stanford Diabetes Research Center (NIH grant P30 DK116074 to SKK) for sequencing support. This work was also supported by NIH awards (R01 DK107507; R01 DK108817; U01 DK123743) to SKK. Work by SK and YH here was also supported by the JDRF Northern California Center of Excellence, grants from the HL Snyder Foundation and gifts from two anonymous donors and from S and M Kirsch.

## Author contribution statement

NK and JAMS designed the research and wrote the original manuscript. NK, YH, NAJK, NP, RC, LR and MP performed the experiments and/or acquired the data. NK, YH and NAJK analysed the data. JAMS, SR, CF and SK supervised the projects. ND and MHS performed tissue sampling for QUOD PANC. GK, DT and YAS provided valuable input on pancreas histology and pancreatic diseases. All authors have reviewed, edited and provided approval on the final version of the manuscript.
