# Supplementary material for: Identification of a vimentin-expressing α-cell phenotype in CF and normal pancreas
Source: J Endocrinol. 2025 Feb 17;264(3):e240190. doi: 10.1530/JOE-24-0190 (PMC11850051; doi:10.1530/JOE-24-0190)
Supplement: Supplementary file 1 [file supplementary_materials.pdf]

Supplementary material

Supplementary Table 1: List of antibody specifications and dilutions

Antibody	Specification	Refe-rence	Lot	Dilu-tion	Company	RRID number
Insulin	Flex polyclonal Guinea Pig, anti-insulin, ready-to-use	IR002	10141925, 11360319, 11590944	1:5	Dako, Agilent Techologies, Cheshire, UK	AB_2800361
Glucagon	Monoclonal Anti-Glucagon antibody produced in mouse, clone K79bB10, Source # 0000078097	G2654-2ML	000080611, 0000175539	1:100	Sigma Aldrich, Merck, Dorset, UK	AB_259852
Vimentin	100 µl (0.268 mg/ml) PUR, Rb mAb to Vimentin [EPR3776]	Ab92547	GR3186827-10,CR325871933, 1072376-1	1:100	Abcam, Cambridge, UK	AB_10562134
Alexa Fluor 647	Alexa Fluor 647 donkey anti-mouse IgG (H+L), 2 mg/ml	A31571	2260928, 2420713	1:500	Invitrogen, Thermo Fisher Scientific, Massachusetts, USA	AB_162542
Alexa Fluor 568	Alexa Fluor 568 goat anti-guinea pig IgG (H+L), 2 mg/ml, highly cross-absorbed	A11075	2160073, 2872022	1:500	Invitrogen, Thermo Fisher Scientific, Massachusetts, USA	AB_2534119
Alexa Fluor 488	Alexa Fluor 488 donkey anti-rabbit IgG (H+L), 2 mg/ml	A21206	2256732	1:500	Invitrogen, Thermo Fisher Scientific, Massachusetts, USA	AB_2535792
Alexa Fluor 488	Goat anti-Rabbit IgG (H+L) Cross absorbed Secondary Antibody, Alexa Fluor 488	A11008	2897813	1:500	Invitrogen, Thermo Fisher Scientific, Massachusetts, USA	AB_143165

Supplementary Table 2: Summary of chronic pancreatitis donor data

ID	Age [year]	Sex	BMI [kg/m^2]	Duration [year]	Group
C01	35	Male	31	2	PD
C02	37	Female	23	7	ND
C03	40	Male	27	17	ND
C04	60	Female	25	17	PD
C05	36	Female	22	20	ND
C06	54	Female	26	15	PD
C07	18	Male	19	1	ND
C08	44	Female	31	4	PD
C09	35	Female	23	5	ND

BMI: Body mass index. Duration denotes years between chronic pancreatitis diagnosis and total pancreatectomy with islet autotransplantation (TPIAT) procedure. PD: pre-diabetes. ND: non-diabetic.

Supplementary Table 4. DEG in vimentin-positive versus vimentin-negative α-cells in CP islets.

Group	Gene	Information
**Cancer associated**	PDK4	Increased malignancy in cancer (1, 2).
	GPX3	Observed in some forms of cancer and is thought to correlate with chemo resistance, cancer progression, and maintenance (3).
	LMAN1	Related to proliferation, migration and invasion of glioma cells (4).
	RP11-124N14.3	Is a long noncoding RNA with the potential to diagnose and predict survival of breast cancer (5). It is positioned antisense to VIM and dysregulated in multiple cancer cells (6).
	PAK6	Overexpressed in prostate cancer and it was shown to impact cell-to-cell connections (7, 8).
	HBB	Upregulated in circulating tumour cells, but not the cancer tissue itself and potentially contributes to their metastatic and invasive features (9, 10).
**Cytokine response**	TNFRSF12A	Receptor binding tumour necrosis factor (TNF)-like weak inducer of apoptosis (TWEAK) and their signalling can impact on fibrogenic responses as well as liver tumour microenvironment (11). The receptor is upregulated in pancreatic cancer and chronic pancreatitis and it was shown to induce invasion and metastasis through EMT activation in cancer cells (11, 12).
	UBE2J1	Plays a role in the recovery of ER stress (13). Can negatively impact on cytokine production (14).
	MAP1B	Microtubule associated protein highly expressed in beta-cells (15).
**Metabolism**	SLC2A13	Is an H^+/myoinositol transporter expressed in endocrine and exocrine (16, 17). Increased mRNA expression in pancreatic tumour tissue (18).
	YWHAZ	Plays role in β-cell function and pancreatic cancer metastasis (19, 20).
	PDZD8	Is a ER-mitochondria binding protein impacting on Ca2+ dynamics (21).

**Cytoskeleton**	EZR	Part of the ezrin-radixin-moesin (ERM) cytoskeletal proteins and is upregulated in pancreatic cancer (22).
	CD44	Is a class I transmembrane glycoprotein which impacts on cell growth, survival, differentiation, motility and tumour metastasis (23, 24).
**Other**	TRBV13	Predicted to be involved in cell surface signalling and to be part of T-cell receptor complex. Acts upstream of or within cytokine-mediated signalling pathway (25).
	TRBV11-3	Predicted to be involved in cell surface signalling and to be part of T-cell receptor complex (26).
	TRBV18	Predicted to be involved in cell surface signalling and to be part of T-cell receptor complex (27).
	TRBV16	Predicted to be involved in cell surface signalling and to be part of T-cell receptor complex (28).
	TRBV12-5	Predicted to be involved in cell surface signalling and to be part of T-cell receptor complex (29).
	TRBV17	Predicted to be involved in cell surface signalling and to be part of T-cell receptor complex (30).
	TRBV7-9	Predicted to be involved in cell surface signalling and to be part of T-cell receptor complex (31).
	IGLV3-27	Predicted to be involved in immune response (32).

References:

1. Dou X, Fu Q, Long Q, Liu S, Zou Y, Fu D, et al. PDK4-dependent hypercatabolism and lactate production of senescent cells promotes cancer malignancy. Nature Metabolism. 2023:1-24.
2. Leclerc D, Pham DNT, Lévesque N, Truongcao M, Foulkes WD, Sapienza C, et al. Oncogenic role of PDK4 in human colon cancer cells. British Journal of Cancer. 2017;116(7):930-6.
3. Chang C, Worley BL, Phaëton R, Hempel N. Extracellular Glutathione Peroxidase GPx3 and Its Role in Cancer. Cancers. 2020;12(8):2197.
4. Du Q, Lin Y, Zhang W, He F, Xu Y, Chen Z. Bioinformatics analysis of LMAN1 expression, clinical characteristics, and its effects on cell proliferation and invasion in glioma. Brain Research. 2022;1789:147952.
5. Wang K, Liao C, Zhong Q, Dong H, Zhang T, Jin R. CeNETs analysis reveals the prognostic value of a signature integration from five lncRNAs in breast cancer. Journal of Cellular Biochemistry. 2019;120(8):13509-19.
6. Kaczkowski B, Tanaka Y, Kawaji H, Sandelin A, Andersson R, Itoh M, et al. Transcriptome Analysis of Recurrently Deregulated Genes across Multiple Cancers Identifies New Pan-Cancer Biomarkers. Cancer Research. 2016;76(2):216-26.

7. Kaur R, Yuan X, Lu ML, Balk SP. Increased PAK6 expression in prostate cancer and identification of PAK6 associated proteins. The Prostate. 2008;68(14):1510-6.
8. Fram S, King H, Sacks DB, Wells CM. A PAK6–IQGAP1 complex promotes disassembly of cell–cell adhesions. Cellular and Molecular Life Sciences. 2014;71(14):2759-73.
9. Zheng Y, Miyamoto DT, Wittner BS, Sullivan JP, Aceto N, Jordan NV, et al. Expression of β-globin by cancer cells promotes cell survival during blood-borne dissemination. Nature Communications. 2017;8(1):14344.
10. Ponzetti M, Capulli M, Angelucci A, Ventura L, Monache SD, Mercurio C, et al. Non-conventional role of haemoglobin beta in breast malignancy. British Journal of Cancer. 2017;117(7):994-1006.
11. Abu Bakar NDB, Carlessi R, Gogoi-Tiwari J, Köhn-Gaone J, Williams V, Falasca M, et al. TWEAK/Fn14 Signalling Regulates the Tissue Microenvironment in Chronic Pancreatitis. Cancers. 2023;15(6):1807.
12. Liu J-y, Jiang L, He T, Liu J-j, Fan J-y, Xu X-h, et al. NETO2 promotes invasion and metastasis of gastric cancer cells via activation of PI3K/Akt/NF-κB/Snail axis and predicts outcome of the patients. Cell Death & Disease. 2019;10(3):162.
13. Elangovan M, Chong HK, Park JH, Yeo EJ, Yoo YJ. The role of ubiquitin-conjugating enzyme Ube2j1 phosphorylation and its degradation by proteasome during endoplasmic stress recovery. Journal of cell communication and signaling. 2017;11:265-73.
14. Feng T, Deng L, Lu X, Pan W, Wu Q, Dai J. Ubiquitin-conjugating enzyme UBE2J1 negatively modulates interferon pathway and promotes RNA virus infection. Virology Journal. 2018;15:1-9.
15. Dorajoo R, Ali Y, Tay VSY, Kang J, Samydurai S, Liu J, et al. Single-cell transcriptomics of East-Asian pancreatic islets cells. Sci Rep. 2017;7(1):5024.
16. Berger C, Zdzieblo D. Glucose transporters in pancreatic islets. Pflügers Archiv - European Journal of Physiology. 2020;472(9):1249-72.
17. Segerstolpe Å, Palasantza A, Eliasson P, Andersson E-M, Andréasson A-C, Sun X, et al. Single-cell transcriptome profiling of human pancreatic islets in health and type 2 diabetes. Cell metabolism. 2016;24(4):593-607.
18. Yang W, Liu H, Duan B, Xu X, Carmody D, Luo S, et al. Three novel genetic variants in NRF2 signaling pathway genes are associated with pancreatic cancer risk. Cancer Science. 2019;110(6):2022-32.
19. Mugabo Y, Zhao C, Tan JJ, Ghosh A, Campbell SA, Fadzeyeva E, et al. 14-3-3ζ Constrains insulin secretion by regulating mitochondrial function in pancreatic β cells. JCI Insight. 2022;7(8).
20. Cao F, Jiang Y, Chang L, Du H, Chang D, Pan C, et al. High-throughput functional screen identifies YWHAZ as a key regulator of pancreatic cancer metastasis. Cell Death & Disease. 2023;14(7):431.
21. Hirabayashi Y, Kwon S-K, Paek H, Pernice WM, Paul MA, Lee J, et al. ER-mitochondria tethering by PDZD8 regulates Ca2+ dynamics in mammalian neurons. Science. 2017;358(6363):623-30.
22. Xu J, Zhang W. EZR promotes pancreatic cancer proliferation and metastasis by activating FAK/AKT signaling pathway. Cancer cell international. 2021;21:1-15.
23. Ponta H, Sherman L, Herrlich PA. CD44: from adhesion molecules to signalling regulators. Nature reviews Molecular cell biology. 2003;4(1):33-45.
24. Avrahami D, Wang YJ, Schug J, Feleke E, Gao L, Liu C, et al. Single-cell transcriptomics of human islet ontogeny defines the molecular basis of β-cell dedifferentiation in T2D. Molecular Metabolism. 2020;42:101057.
25. Alliance of Genome Resources. TRBV13 2022 [Available from: https://www.alliancegenome.org/gene/HGNC:12188.
26. Alliance of Genome Resources. TRBV11-3 2022 [Available from: https://www.alliancegenome.org/gene/HGNC:12182.
27. Alliance of Genome Resources. TRBV18 2022 [Available from: https://www.alliancegenome.org/gene/HGNC:12193.

28. Alliance of Genome Resources. TRBV16 2022 [Available from: https://www.alliancegenome.org/gene/HGNC:12191.
29. Alliance of Genome Resources. TRBV12-5 2022 [Available from: https://www.alliancegenome.org/gene/HGNC:12187.
30. Alliance of Genome Resources. TRBV17 2022 [Available from: https://www.alliancegenome.org/gene/HGNC:12192.
31. Alliance of Genome Resources. TRBV7-9 2022 [Available from: https://www.alliancegenome.org/gene/HGNC:12243.
32. Alliance of Genome Resources. IGLV3-27 2022 [Available from: https://www.alliancegenome.org/gene/HGNC:5910.
